# Noncoding RNA Regulation of Hormonal and Metabolic Systems in the Fruit Fly *Drosophila*

**DOI:** 10.3390/metabo13020152

**Published:** 2023-01-19

**Authors:** Ki-Kei Chan, Ting-Fung Chan, William Bendena, Jerome H. L. Hui

**Affiliations:** 1 State Key Laboratory of Agrobiotechnology, School of Life Sciences, The Chinese University of Hong Kong, Hong Kong SAR, China; 2 Simon F.S. Li Marine Science Laboratory, School of Life Sciences, The Chinese University of Hong Kong, Hong Kong SAR, China; 3 Institute of Environment, Energy and Sustainability, The Chinese University of Hong Kong, Hong Kong SAR, China; 4 Department of Biology, Queen’s University, Kingston, ON K7L 3N6, Canada

**Keywords:** ncRNA, miRNA, lncRNA, circRNA, *Drosophila*, hormone, endocrinology, metabolism

## Abstract

The importance of RNAs is commonly recognised thanks to protein-coding RNAs, whereas non-coding RNAs (ncRNAs) were conventionally regarded as ‘junk’. In the last decade, ncRNAs’ significance and roles are becoming noticeable in various biological activities, including those in hormonal and metabolic regulation. Among the ncRNAs: microRNA (miRNA) is a small RNA transcript with ~20 nucleotides in length; long non-coding RNA (lncRNA) is an RNA transcript with >200 nucleotides; and circular RNA (circRNA) is derived from back-splicing of pre-mRNA. These ncRNAs can regulate gene expression levels at epigenetic, transcriptional, and post-transcriptional levels through various mechanisms in insects. A better understanding of these crucial regulators is essential to both basic and applied entomology. In this review, we intend to summarise and discuss the current understanding and knowledge of miRNA, lncRNA, and circRNA in the best-studied insect model, the fruit fly *Drosophila*.

## 1. Introduction

The fruit fly *Drosophila melanogaster* is an important model organism used in biological research and is arguably the most extensively studied insect genus. Similar to other holometabolous insects, it has a life cycle enduring multiple morphological transitions—embryos, larvae, pupae, and adults [1]. Accompanying these life stage transitions, alterations in the metabolic system due to hormonal fluctuations result in the turnover of the cell population within the *Drosophila*’s body. In general, *Drosophila* is considered to have three instar larval stages determined by molting. During molting, the larvae shed a layer of the cuticle as a fresh layer replaces the previous layer modulated by hormones, a process known as ecdysis. Subsequently, the third instar larvae will transform into pupae and eventually emerge as adults. Two hormonal systems are responsible for modulating the entire process of development and metamorphosis: the sesquiterpenoid and the ecdysteroid systems [1,2,3,4,5]. In this review, we will discuss the established roles of non-coding RNAs (ncRNAs) in *Drosophila* and how select ncRNAs have been identified that regulate hormonal and metabolic pathways.

## 2. microRNAs in *Drosophila*

ncRNAs are a large and diverse class of RNA molecules that do not encode proteins, and their essential roles have only been recognised within the last few decades (Brandenburger et al., 2018 [6]). These include functional ncRNAs such as ribosomal RNA (rRNA) and transfer RNA (tRNA) involved in the protein translation process [7], and others with regulatory effects such as miRNAs and lncRNAs. MiRNAs are processed from larger double-stranded RNA precursors to create RNA molecules of 21–23 nucleotides. In contrast, lncRNAs are longer, more than 200 nucleotides long. With their differences in length, miRNA and lncRNA utilise different mechanisms to regulate the expression of their target genes. Moreover, lncRNA can bind with proteins forming a scaffold of ribonucleoprotein complexes [8] or function as a competing endogenous RNA that serves as a decoy for miRNA to bind to, eventually inhibiting miRNA regulatory effects [9]. In this section, we will first discuss the miRNAs which are summarized in Table 1.

MiRNAs are relatively conserved between bilaterians and cnidarians [10,11,12]. As one of the small ncRNA classes, primary miRNAs are transcribed from DNA and further processed into preliminary miRNAs by enzymes such as Drosha in the canonical miRNA biosynthesis pathway [13]. Preliminary RNAs are hairpin structures cleaved by Dicer and its cofactor Loquacious resulting in the formation of miRNA/miRNA duplexes. The miRNA/miRNA duplexes will be loaded by Argonautes (Ago), and one of the duplex strands will be retained to form a miRNA-induced silencing complex (miRISC). The miRISCs interact with the 3′ untranslated regions (UTRs) of the mRNAs by complementary binding, resulting in the transcriptional repression of the target mRNAs [2,3,14,15,16]. Specific mechanisms of transcriptional repression vary due to the utilization of different Ago proteins in the miRISC. Ago1-RISC represses the translation after 5′cap recognition, and Ago2-RISC blocks the recognition directly [15]. Previous studies also suggested that miRNAs are also capable of binding to open-reading frames (ORF) and 5′UTR [17,18]. Under specific circumstances, miRNA binding can induce mRNA expression by binding to the promoter sites or binding to the 5′TOP motif of rRNA to alleviate translational repressions [19,20,21]. In the *Drosophila* genome, 258 miRNAs have been annotated (miRBase Release 22.1) [22].

### 2.1. miRNA in Drosophila Development and Metamorphosis

Some miRNAs, such as miR-34, are maternally inherited in *Drosophila* and can regulate early embryonic development [23]. In a study knocking out ~130 microRNAs, several miRNAs were found to be essential for survival to adulthood, including *bantam*, miR-1, the let-7 cluster, miR-8, the miR-309 cluster, miR-263a, and miR-276a [24]. Among the 1065 maternal transcripts, 710 were identified as targets for the SMAUG protein. An additional 92 transcripts were regulated by miR-309 and SMAUG, which cooperatively monitor the degradation of maternal mRNA and the maternal-to-zygotic gene expression transition [25,26,27,28].

During *Drosophila* embryonic development, miRNAs regulate the apoptotic process [29]. By binding to the 3′UTR of mRNAs expressing *reaper* (*rpr*), *grim*, *head involution defective* (*hid*), and *sickle* (*skl*), miR-6 and miR-11 are shown to regulate embryonic apoptosis, and a double knock-out of miR-6/miR-11 induces defects in the central nervous system [29]. Another miRNA *bantam* also functions in the CNS scaling the growth of Class IV dendrite arbors in *Drosophila* sensory neurons in the late embryonic to early larval stages [30]. In larval development, miR-281, miR-311, miR-79, miR-92, miR-305, miR-131, and miR-31a were predicted to target genes involved in this process [31].

In addition to miRNA involvement in metamorphosis, miRNAs have been suggested to regulate gene expression in forming adult wing structures [32]. In *Drosophila*, let-7, miR-125, miR-1, miR-2b, miR-2c, miR-9a, and miR-13b control wing development [32,33,34,35], while loss of miR-987 function resulted in indirect flight muscle defects in an age-dependent manner [35]. Another morphogenesis aspect during the adult stage is the abdomen where miR-965 regulates *string* (*stg*) and *wingless* (*wg*) mRNAs to control its cell proliferation and migration [36], and leg development is controlled by a microRNA cluster miR-6/5/4/286/3/309 [37].

**Table 1:** Summarisation of miRNAs reported with their validated targets, function, and hormonal and metabolic regulations according to their references.

miRNA	Validated Targets	Function, Hormonal and Metabolic Regulations	References
Development and Metamorphosis			
miR-6, miR-11	*rpr*, *grim*, *hid*, *skl*	Embryonic apoptosis and CNS development	[29]
*bantam*	*Akt*	Dendrite arbour growth	[30]
miR-281, miR-311, miR-79, miR-92, miR-305, miR-131, miR-31a	N/A	Larval development	[31]
let-7, miR-125	N/A	Wing development	[33]
miR-9a	*dLMO (Beadex)*	Wing development	[34]
miR-1, miR-2b, miR-2c, miR-13b, miR-987	N/A	Wing development	[35]
miR-965	*stg*, *wg*	Abdomen morphogenesis	[36]
miR-6/5/4/286/3/309	N/A	Leg development	[37]
miR-277, miR-304	*mbl*	Muscle development	[38]
Sesquiterpenoid and Ecdysteroid system			
*bantam*	*JHAMT*	JH biosynthesis	[39]
miR-252, miR-304	*JHAMT*	JH biosynthesis	[39]
miR-8, miR-14, miR-34, miR-278	*Met/Gce*	JH signalling pathway	[39]
miR-927	*Kr-h1*	JH signalling pathway	[40]
*bantam*	*phantom, shade, disembodied*	Ecdysone biosynthesis	[41]
miR-14	*EcR*	Ecdysone signalling pathway	[42]
let-7, miR-125	*dpt*	Regulated by ecdysone; innate immune systems	[43]
miR-8	N/A	Cell growth regulated by ecdysone; induce JH biosynthesis pathway	[44]
miR-252	Abi	Cell division	[45]
Insulin pathway and Lipid metabolism			
miR-8	*ush*	Insulin signalling pathway; body size	[46]
miR-7	*cpa*	Insulin secretion pathway	[47]
miR-9a	*short neuropeptide F receptor 1*	Insulin signalling pathway; body size	[48]
miR-14	*sg*	Insulin-producing pathway; fat accumulation	[49]
miR-305	*Dp53*	TOR regulator signalling in response to nutrient	[50]
miR-305	*InR, PI3K, Hairless*	Notch and insulin pathways in adaptive homeostasis	[50,51]
miR-277	*FAO*	Fatty acid metabolism; homeostasis	[52]
miR-14	N/A	Fatty acid metabolism	[53]
miR-278	*ex*	Insulin sensibility	[54]
Sexual Development			
miR-184	*saxophone*	Female germline development; nurse cell nutrient support; Egg chamber size	[55]
miR-318	*Tramtrack69*	Oogenesis; chorion gene amplification	[56]
miR-282	*rut*	Egg production; apoptotic activity	[57]
miR-989	N/A	Border cell migration in ovaries	[58]
*bantam*	N/A	Adult germline stem cell formation	[59]
*bantam*	*dFmr1*	GSCs maintenance in ovaries	[60]
miR-7, miR-309, miR-278	*dap*	Cell cycle regulations	[61]
let-7	N/A	Sex-biased ecdysone signalling; aging in testis stem cells and fertility	[62,63]
miR-190	N/A	Sexually dimorphic in male; regulating neuronal activities and lifespan	[64]
Lifespan and Aging			
*bantam*, miR-1, miR-190, miR-279, miR-996	N/A	Survival; viability	[24]
miR-282	N/A	Increase lifespan	[57]
miR-305	N/A	Aging; locomotor activity; abnormal protein aggregation in muscle; oxidative stress	[65]
miR-184, let-7	N/A	Prolonged lifespan	[66,67]
miR-125	*Chinmo*	Prolonged lifespan	[68]
miR-34	*Pcl*, *Su(z)12*	Chaperone expressions; healthy brain aging	[69,70]
miR-34	*Lst8*	Healthy brain aging	[71]
miR-277	N/A	Branched-chain amino acid catabolism; growth regulation; Reduced lifespan	[72]
Circadian Rhythm and Photoperiod			
miR-124	BMP signalling	Circadian rhythm; rhythmic normality	[73]
miR-276a	*tim*	Circadian rhythm	[74]
miR-279	*unpaired*	Circadian behaviour; JAK/STAT circuit	[75]
miR-959-964	N/A	Rhythmic feeding; loop of feeding period control	[76]
miR-210	*Fasciclin 2*	Photoreceptor function	[77]
miR-263a, miR-263b	*clk*, *cwo*	Circadian rhythm	[78]
let-7	*cwo*	Circadian rhythm	[79]

### 2.2. miRNA in the Sesquiterpenoid and Ecdysteroid Systems

The entire process of metamorphosis in insects is initiated and regulated by two hormonal systems, namely the sesquiterpenoid and the ecdysteroid hormone systems [2,3,4,80]. The corpus allatum (CA) in the ring gland of *Drosophila* produces three forms of sesquiterpenoid hormones: juvenile hormone III (JH III), juvenile hormone bisepoxide (JHB3), and methyl farnesoate (MF). Juvenile hormones induce the expression of specific genes such as *Krüppel homolog 1* (*Kr-h1*). The signalling network results in the inhibitory effect of these genes towards the molting process and postpones the initiation of metamorphosis, prolonging the juvenile characteristics of the larvae.

The prothoracic gland (PG) in the ring gland produces ecdysone through the ecdysteroid biosynthesis pathway by enzymes expressed from a set of *Halloween* genes. Ecdysone is converted into 20-hydroxyecdysone (20E) by the enzyme encoded by *shade* in the peripheral tissues, such as the fat body [81,82]. Ecdysones are essential for the transition of signalling pathways utilising the transcription factor Chronologically Inappropriate Morphogenesis (Chinmo) to Broad/E93 transcription factors [83]. Williams and Kafatos brought up a theory fifty years ago, suggesting there may be three “master regulatory genes” that can reciprocally inhibit each other, and each will be responsible for regulating a set of “stage-specific genes” in the larval, pupal, and adult stages [84]. Later findings disputed the theory of stage-specific genes since many genes are responsible for metamorphosis and may be expressed in more than one stage. Referencing this theory, Truman and Riddiford found the three “master regulatory” transcription factors Chinmo, Broad, and E93 reciprocally inhibit each other, and each of them induce the expression of different downstream genes throughout metamorphosis due to the induction of JH and 20E [85]. However, the ecdysteroid turnover remains obscure for many insects, including *Drosophila*. It has been established that the ecdysteroid variations may differ in *Drosophila* due to diet and development [86]. Regardless of the unknown biosynthesis mechanism, it is well-acknowledged that the two types of hormones collaboratively regulate the proceeding of life stages and control larval molting or onset of metamorphosis in arthropods via binding to their respective receptors [87]. The different levels of antagonistic hormones within the ring gland determine the developmental stages’ transition [88]. Both types of hormones yield the potential as transcription activators and inhibitors, elevating or repressing their target’s transcription level. Some have argued that JH stimulates the *Broad-Complex* (*Br-C*) in hemimetabolous insects, and through the evolution to holometabolan insects, JH became inhibitory to the *Br-C* transcripts [89]. The repression of *Br-C* by JH in holometabolan insects is well-established and indisputable [90,91,92].

MiRNA can regulate the JH pathway and the ecdysone pathway by binding to the transcripts of protein-coding genes in the process of hormone biosynthesis and the downstream signalling pathways [39,41]. Given that the hormonal pathway of *Drosophila* regulates the development and onsets of metamorphosis, evaluating miRNA regulation on the hormonal pathways would be critical for understanding insect development.

The miRNA expression levels differ among developmental stages in *Drosophila* [93,94], suggesting these differentially expressed miRNA may be correlated with development and metamorphosis. Many enzymes catalyze the sesquiterpenoid biosynthetic pathway, and the juvenile hormone acid O-methyltransferase (JHAMT) is responsible for the rate-determining step [3]. The *JHAMT* expression level determines the JH titre, and *DmJHAMT* knockdown mutant males have disoriented male genitals [95]. Overexpression of microRNA *bantam* resulted in similar phenotypes [39]. In addition, in *bantam* overexpression mutants, JHIII and JHB3 titres were significantly reduced [39]. Moreover, in vitro dual luciferase reporter assay and pull-down assay verify the interaction of *bantam* and *JHAMT* mRNA transcript, confirming the regulation of *bantam* in the sesquiterpenoid pathway. Furthermore, in vitro experiments demonstrate the potential miRNA candidates for regulating the expression of *JHAMT* mRNA and the JH receptors *Methoprene-tolerant* (*Met*) and *Germ cell expressed* (*Gce*) mRNA. MiRNA candidates targeting the *JHAMT* transcript may include *bantam*, miR-252, and miR-304, and the miRNA candidates targeting the *Met*/*Gce* transcript include miR-8, miR-14, miR-34, and miR-278 in *Drosophila* [39].

Interestingly, *bantam* inhibits ecdysone production by indirectly regulating some of the Halloween-gene-expressed mRNAs, encoding the enzymes for the ecdysteroid biosynthesis pathway [41]. Boulan et al. suspects the high *bantam* level was for maintaining lower ecdysone titre and promoting growth, while the lowered *bantam* level at the third instar larvae stage is for the surge of ecdysteroids for entering the metamorphic transitions [41].

**Figure 1:**
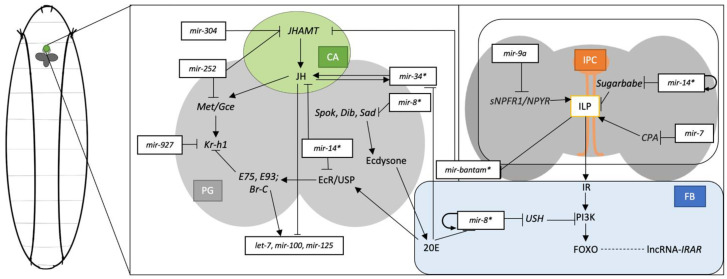
*Drosophila* larval hormonal signalling systems and metabolic systems represented with the juvenile hormone (JH), ecdysone, and insulin in the ring gland (RG), insulin-producing cells (IPC) from the brain, and adipose cells from fat body (FB). The RG is represented with corpus allatum (CA) coloured in green, prothoracic gland (PG) in light grey, IPCs in orange, the brain in dark grey, and fat body (FB) in blue. MiRNAs regulation on the specific pathways are represented throughout the pathways. Hormonal crosstalk involves miR-34*, miR-8*, bantam*, and miR-14* as they interplay in multiple systems.

As JH binds to the Met/Gce receptor, the complex will initiate the transcription of Kr-h1. It is worth noting that miR-927 can bind to the transcript of Kr-h1 and down-regulate its expression in *Drosophila* [40], and its mutants exhibit similar traits to the Kr-h1 mutants [96]. It has been previously established that miR-14 has the potential to regulate the JH system via the Met/Gce receptor. There have also been reports on the regulatory role of miR-14 in ecdysteroid systems. miR-14 knock-out mutants have overexpression of ecdysone receptor (EcR) transcripts and downstream gene transcripts [97]. These two findings suggest that miR-14 may be one of the key regulators in hormonal systems, as shown in Figure 1.

As described, miRNAs may have regulatory effects on the sesquiterpenoid and ecdysteroid systems, and vice versa, since the two systems also induce the expression of miRNAs. Similarly, in *Drosophila*, let-7 and miR-125 are co-expressed during metamorphosis, and mutants of these miRNAs also experience defects throughout metamorphosis [33]. The expression of let-7, miR-125, miR-100, and miR-34 can also be observed during the *Drosophila* development stages, each occurring according to specific hormones [40,41]. The let-7, miR-100, and miR-125 cluster expressions are enhanced by the introduction of ecdysone, whereas the expression of miR-34 is strictly inhibited. Meanwhile, the expression level of miR-34 is enhanced by the introduction of JHs, but the let-7, miR-100, and miR-125 are strictly inhibited [41]. Since Qu et al. [39] demonstrated the potential inhibitory effect of miR-34 on *Met*/*Gce*, miR-34 can regulate the sesquiterpenoid pathway through feedback inhibition. Br-C knock-out mutants significantly reduce the RNA level of let-7, miR-100, and miR-125. Br-C is crucial for the up-regulation of let-7, miR-100, and miR-125. Eventually, by introducing JH and ecdysones separately, the corresponding miRNA transcript levels increase or decrease accordingly. Thus, JH induces the transcription of miR-34, and ecdysteroid enhances the transcription of let-7, miR-100, and miR-125 via the Br-C [41,42].

Ecdysone-induced miRNA transcription can further regulate other pathways. Let-7 and miR-125 were validated for their regulatory effects on innate immune systems, with let-7 regulating the antimicrobial peptide gene *diptericin* (*dpt*) [43]. Conserved ecdysone-induced miR-8 regulates the insulin signalling pathway by targeting the *u-shaped* (*ush*) transcript, and the overexpression of miR-8 will result in mutants with larger body sizes [98]. Moreover, enhanced miR-8 has been found to promote cell growth in the CA and elevation in the juvenile hormone biosynthesis pathway independently from the insulin signalling pathway [44]. Apart from the inhibitory effect of miR-252 on *JHAMT* transcripts, ecdysone-responsive miR-252 controls the cell cycle of *Drosophila* by targeting the Abelson interacting protein (Abi), suggesting more cell division is occurring through metamorphosis due to the presence of miR-252 [45,94].

Even though many regulatory effects of miRNAs on hormonal systems have been revealed and validated, many potential miRNA-mRNA interactions remain in predictions. The miRNA target predictions are primarily established through the algorithm estimating the stability of annealed complementary pairing of seed regions of miRNAs and mRNAs [99,100]. Certain regions of the transcript sequences may yield a conserved site for miRNA-mRNA interaction. These predictions suggest that some downstream genes of the ecdysteroid system interact with specific miRNAs [31]. Furthermore, the ecdysone-induced miRNA differentially expressed profile with 72 distinct miRNAs was identified with or without 20E [101]. More tools utilising different algorithms are available for more advanced predictions as well. These findings suggest that the current understanding of potential miRNA regulation on the sesquiterpenoid and ecdysteroid pathways is only the tip of the iceberg. The unrevealed modulation will be necessary for a wholesome understanding of the *Drosophila* endocrine system. Future investigation may also focus on miRNA regulation of hormonal production due to neuropeptide signalling of the ring gland for understanding the crosstalk in the endocrine systems of *Drosophila* [102].

### 2.3. Insulin Pathway and Lipid Metabolism

The crosstalk between the hormonal and metabolic pathways are exceptionally sophisticated, as exemplified by the insulin and ecdysteroids signalling pathways. [2]. The previous section mentioned that miR-8 regulates the insulin pathway through *ush* with the induction of ecdysteroids [98]. More specific to the insulin pathway, the Drosophila insulin-like peptides (dILPs) bind to the insulin receptors (IR) and trigger the downstream phosphorylating cascade involving components such as chico, phosphoinositide-3 kinase (PI3K), and Akt kinase [103,104,105,106]. Since miR-8 regulates the *ush* transcript in fat cells, miR-8 null mutants suffer from increased brain cell apoptosis and some flies have malformed legs and wings [46]. miR-8 and *ush* can regulate insulin receptor expression level through the PI3K pathway [46]. miR-7 regulates the secretion of insulin-like peptides (ilp) by targeting the F-actin capping protein alpha (CPA) [47]. On the other hand, insulin signalling represses *bantam* transcription through dILP regulations, interfering with the ecdysteroid system as well [95].

Additional miRNAs are responsible for insulin pathway regulations and energy metabolism. Insulin-producing cells are regulated by *miR-9a* and by body sizes [48]. Insulin production from insulin-producing neurosecretory cells is regulated by miR-14 through the regulation of *Sugarbabe* (*sug*), which is directly related to the insulin-producing pathway [49]. Overexpression of *sug* results in fat accumulation and reduces the insulin-like peptide’s mRNA expression level. Unlike *sug*, miR-14 does not seem to be controlled by nutritional status and may play a part in insulin production and fat metabolism maintenance [49]. The *Drosophila* homolog of *p53* (*Dp53*) is regulated by miR-305 in a nutrient-dependent matter [50]. The expression level of miR-305 varied between well-fed and nutrient-deprived animals, and the system is induced by TOR regulator signalling [50]. miR-305 was found to regulate the Notch and insulin pathways in intestinal stem cells of *Drosophila* [51]. Through these two pathways, Foronda et al. also presented the potential of miR-305 in mediating adaptive homeostasis in the *Drosophila* gut [51]. Similarly, miR-277 regulates fatty acid β-oxidation (FAO) enzymes in intestinal stem cells. The differentially expressed genes are identified in miR-277 mutants [52]. Validations suggested disrupting homeostasis and progenitor cell survival with miR-277 mutants [52]. The alteration of fat metabolism by miR-14 suggested that the triacylglycerol and diacylglycerol levels differ in miR-14 mutants [53].

In the insulin signalling pathway, miR-278 targets *expanded* (*ex*) to sustain insulin sensibility [54]. Interestingly, miR-278 is abundantly expressed in wild-type flies, keeping the *ex* mRNA at lower levels in the adipose tissue, unlike the minute dosage of many other miRNAs [54]. Such abundant expression of miR-278 could be an example of solid modulation of miRNAs on its target transcript, proving the importance of its regulation in a cellular system.

### 2.4. miRNA in Sexual Development

Larval ovary morphogenesis in *Drosophila* is closely related to the ecdysone, insulin, Activin, Dpp, and EGFR signalling pathways. It has also been reported that the miRNA pathway monitors Drosophila larval ovary morphogenesis [107]. Sexual development and maturation in *Drosophila* are monitored and determined by miRNAs. Reports on miR-184 in female germline development reveal the roles it plays [55]. Mutant females are reduced in size and may fail to develop functional nurse cells due to the lack of oocyte nutrient support. This results in the reduced egg chamber size in females [55].

Similarly, miR-318 has multiple roles during oogenesis. Ecdysone signalling pathways induce the expression of miR-318, and miR-318 interferes with the follicle cells’ involvement in chorion gene amplifications to synthesize the eggshell structure [56]. miR-282 is associated with egg production due to elevated apoptotic activity in mutant mir-282 ovaries, possibly due to the increased *rutabaga* (*rut*) adenylate cyclase gene targeted by mir-282 [57]. Another study revealed that border cell migration in the ovary requires the modulation of miR-989. Mutants without miR-989 displayed migration of border cells in mutant egg chambers for the somatic cells but not in the germline cells [58].

The *Bantam* loss-of-function mutant resulted in defects of adult germline stem cells (GSC) [59]. *Bantam* is also associated with the *Drosophila* ortholog of Fragile X mental retardation protein (*dFmr1*) in regulating GSCs maintenance and sustaining GSCs within Drosophila ovaries [60]. Dacapo (dap) is a cyclin-dependent kinase inhibitor that monitors the cell cycle in the GCS. miR-7, miR-309, and miR-278 directly regulate the *dap* 3′UTR. Among them, miR-7 and miR-278 may affect the cell cycles in GCSs as mir-7 and mir-278 knock-out mutants have reduced division kinetics and a reduced number of progeny cells [61]. However, not only do miR-7, miR-278, miR-309, and *bantam* serve the sexual development in *Drosophila*, but the monitoring enzymes of miRNA biosynthesis, Dicer and Pasha, are required for ovary morphogenesis [60]. Yang et al. identified the miRNAs that are differentially expressed in the Drosha mutant [60], including let-7, miR-2, miR-8, miR-14, miR-33, miR-125, miR-184, and miR-277. Nevertheless, the differentially expressed genes in the germline of ovariole morphogenesis have been identified by Tarikere et al. [108]. The target genes of the mentioned miRNAs may be differentially expressed and identified for future studies.

Among miRNAs, let-7 determines sexual identity in male-specific regulation of the X chromosome. There is a sex-biased ecdysone signalling where let-7 plays a key role as modulator [62]. Let-7 also contributes to the regulation of aging in testis stem cells, regulating the fertility of *Drosophila* [63]. However, miRNAs not only regulate gonadogenesis and the morphological development of the two sexes, but they may also be expressed in a sexual dimorphic manner. Fernandes and Varghese [64] found that miR-190 shows higher expression levels in male flies and maintains neuronal activities and lifespan. This finding suggests that the miRNA expression level differences in males and females may be due to other regulatory differences that are worth further investigation.

### 2.5. miRNA in Drosophila Lifespan and Aging

Apart from the miRNAs mediating cell death, some miRNAs are associated with lifespan and longevity, and other specific miRNAs result in reduced viability. For instance, *bantam*, miR-1, miR-190, miR-279, and miR-996 were reported to be essential for survival, and the loss of these miRNAs resulted in reduced viability [24]. As previously mentioned, miR-282 regulated egg production in sexual development. miR-282 knock-out mutants have shortened lifespans by 50% on average [57]. miR-305 regulates the aging effect, and miR-305 expression levels decrease through aging. It has also been shown that miR-305 is correlated with locomotor activity, abnormal protein aggregation in muscle, and oxidative stress [65]. In *Drosophila*, miR-125, miR-184, and let-7 alter *Drosophila* lifespan [66,67]. It is first known to have reduced effects on lifespan with let-7 down-regulated mutants, and, following such findings, let-7 overexpression resulted in a prolonged lifespan [66,67]. miR-125 and let-7 expression is elevated in response to dietary restriction, thus extending lifespan. miR-125 regulates the nutrient-dependent transcription factor Chinmo with dietary restriction and restrains the downstream genes of Chinmo from expressing to increase the lifespan in flies [68]. These findings confirm miRNA impacts on increased lifespan and the importance of dietary restrictions.

miR-277 and miR-34 from the same cluster have been reported to interfere with lifespan. miR-277 has been known to control branched-chain amino acid catabolism and further regulated growth regulator TOR kinase. miR-277 expression reduced lifespan, especially on protein-rich diets [72]. On the other hand, miR-34 was associated with healthy brain aging and extended lifespan [69]. miR-34 was proven to regulate *Pcl* and *Su(z)12* mRNA, resulting in higher chaperone expressions [70]. *Lst8* is also regulated by miR-34 and has proven to be differentially expressed in mutant animals [71]. Altogether, miR-34 maintains proteostasis progress and results in healthy brain aging.

### 2.6. miRNA in Drosophila Circadian Rhythm and Photoperiod

Circadian rhythm in animals can be linked to locomotor activity and feeding behavior, sleep/wake patterns, various physiological and metabolic pathways, and even lifespan [109,110]. The circadian rhythm in *Drosophila* is moderated by the circadian feedback regulatory loop composed of the *clock* (*clk*), *cycle* (*cyc*), *period* (*per*), and *timeless* (*Tim*) genes in circadian neurons [111]. Kadener et al. suggested that the 3′UTR can be regulated through miRNAs and the miRNA biosynthesis pathway [112]. As a loss of function in the *per* mutant will result in an alteration in lipid metabolism and increased susceptibility to starvation with lack of nutrients, it is likely for many metabolic pathways to be altered due to circadian rhythm alterations [113]. miR-276a and miR-124 modulate the circadian rhythm, and mutants exhibit behavioural arrhythmicity [73,74]. miR-279 regulates circadian behaviour through the JAK/STAT circuit for cell signalling [75], while miR-996 regulates rhythmic behaviour [114]. Other than that, the miR-959-964 cluster is determined by rhythmic feeding and forms a loop with feeding period control [76]. Locomotor rhythm is regulated by miR-210 through *Fasciclin 2*, a cell adhesion molecule possibly associated with photoreceptors [77]. Analysis of the miRNA expression profile due to photoperiod reveals miR-2b, miR-11, miR-34, miR-274, miR-184, and miR-285 [115]. These miRNAs may also be associated with circadian rhythm.

Several miRNAs are differentially regulated when flies are put in constant darkness compared to the light-dark cycle [78]. Through target site predictions, miR-263a and miR-263b are predicted to regulate *clk* and a circadian rhythm transcription repressor *clockwork orange* (*cwo*), as some other miRNAs were identified and predicted to interfere with the circadian rhythm [78]. Let-7 is proven to repress the expression level of *cwo*. Since circadian prothoracicotropic hormone acts as a transcription factor to initiate ecdysteroid signalling, a regulatory circuit of the circadian cycle consisting of the ecdysteroid system and let-7 is established [79]. It is also worth noting that let-7, miR-375, miR-92a, and *bantam* are identified as regulating sleep and sleep homeostasis in multiple ways [116,117,118,119,120].

Loss of fragile X mental retardation protein (dFmr) is also associated with altered circadian rhythms and leads to altered expression of miR-1 and miR-281 in fragile X syndrome [110]. It is also suggestive that miR-1 and miR-281 can be associated with circadian rhythm.

## 3. lncRNA in *Drosophila*

LncRNAs are RNA molecules with more than 200 nucleotides in length that can function as signals, decoys, guides, and scaffolds [121]. Most lncRNAs are transcribed through the RNA polymerase II (Pol II) with a 5′ cap and 3′ poly-A tail [122]. Even though many lncRNA functions remain unknown, the complexity of lncRNAs in *Drosophila* is not dismissible for their potential roles in transcriptional activation and inhibition [123]. LncRNAs can interfere with mRNA stability and transcription levels, mRNA splicing, and protein activities, and they act as small RNA precursors or sponges [123]. For instance, the Drosophila long non-coding heat-stress-inducible hsrω transcripts appear to monitor protein synthesis status and are developmentally regulated under non-heat shock conditions [124,125]. The lncRNAs that are discussed in this section are summarised in Table 2. Conservation of lncRNAs across species is still noticeable, with many lncRNA orthologues identified between flies, mice, and humans [126].

### 3.1. lncRNA in Development, Metamorphosis, and Ecdysteroid Hormone Systems

In *Drosophila* embryogenesis, *intraabdominal* (*iab*) is known for regulation of the homeotic (*Hox*) transcription factors. *Ultrabithorax* (*Ubx*), *Abdominal-A* (*Abd-A*), and *Abdominal-B* (*Abd-B*) are known for the spatiotemporal expression pattern in development and its mutants will have disrupted abdominal segments [127]. However, for *lncRNA:PS4*, despite having the same expression profile as *Ubx*, promoter deletion in the *lncRNA:PS4* mutant shows no direct expression difference in *Ubx* [140]. Another lncRNA, *acal*, is shown to be responsible for dorsal closure through the JNK signalling pathway, and mutations in *acal* are lethal [128].

LncRNAs that are differentially expressed throughout metamorphosis have been identified previously. Among all, 21% and 42% lncRNAs were significantly up-regulated at late-embryonic and late-larval stages, respectively, suggesting a drastic change in transcriptome throughout different life stages [141]. For example, leg development in *Drosophila* is dependent on *lncRNA:CR33938* and a mutant of this lncRNA results in alteration of expression level in leg development genes [129]. Relevant to the metamorphosis process that is regulated by the ecdysone signalling pathways, a study on the hormone response network for ecdysone reveals four ecdysone-induced lncRNAs: *CR43432*, *CR43626*, *CR45391*, and *CR45424*, without any further analysis [142].

### 3.2. lncRNA in Nutrient Metabolism and Aging

Metabolism can be altered due to many reasons, and one is infection-induced alteration. The lncRNA *IBIN* is overexpressed in response to bacterial infection [130]. The induction of *IBIN* results in the up-regulation of carbohydrate metabolism gene cluster expression and the down-regulation of the amino acid metabolism gene cluster [130]. Such findings suggest that transcriptomic regulation of the immune system and the modulation of pathway preferences in energy metabolism can be regulated by lncRNAs. Another study suggests that with changes in environmental nutrition, lncRNA-*IRAR* mutants of overexpression are more sensitive to the changes and CRISPR-Cas9 mutants are less sensitive [131]. The underlying mechanism has been identified as the insulin receptor transcript expression level due to the similar expression pattern through FOXO binding in the insulin pathway [131].

The environmental nutrient affects the aging process through dietary restriction. The expression profile of flies with dietary restrictions and fully fed flies identifies 102 differentially expressed lncRNAs and 1406 differentially expressed mRNAs, suggesting the potential roles of these transcripts may be differentially expressed due to dietary restrictions. The differentially expressed transcripts are annotated with aging-related signalling pathways in the GO and KEGG databases [143].

### 3.3. lncRNA in Sexual Development

Many annotated lncRNAs have been identified as playing roles in sexual development. Integrated data with RNA-seq and ChIP-seq identified the expressed mRNA and lncRNA throughout the *Drosophila melanogaster* lifespan revealing a high level of lncRNAs in pupal stages and adult males in comparison to adult females, suggesting the expression profile differences in sexes for lncRNA expression profiles [132]. The conservation of lncRNAs in the *Drosophila* genus reveals that trends observed in *Drosophila melanogaster* may not be followed by *Drosophila pseudoobscura*. Both species have shown sex differences in lncRNA expression profiles to be more abundant in males for male development. However, the pupal lncRNA expression profile elevation was not observed in *D. pseudoobscura* [144].

The lncRNA expression profile shows excess lncRNA expression in adult males within days of emergence, potentially associated with adult male sexual development [132]. *Male-specific abdominal* (*msa*) lncRNA is required for accessory gland development in males. The MSA transcript overlaps with lncRNA *iab-8*, both embedded with *miR-iab-8*. The lncRNA *iab-8* and the miRNA embedded with *miR-iab-8* have a regulatory effect on *Hox* genes, whereas the lncRNA *msa* regulates secondary cell morphology and male fertility [133]. The two different lncRNAs, even though embedding the same miRNA, have different regulatory effects on different tissues [133]. This difference is caused by the micro-peptides encoded within the small ORF within the lncRNA *msa* transcript, demonstrating the potential for micro-peptide encoding lncRNAs [145].

The lncRNAs *roX1* and *roX2* are associated with the male-specific lethal (MSL) protein complex, and double *roX* gene knock-out mutants are lethal in males, suggesting the essential role of the *roX* genes [134]. The formation of the MSL protein complex and *roX* genes will increase X-linked gene transcription, resulting in a hyperactive X chromosome in males [135]. Since females have two pairs of X chromosomes, the dosage compensation effect is not required. Therefore, roX gene expression is only detected at the initial blastoderm stage in embryos [146]. Furthermore, the processes of spermatogenesis and oogenesis have been reported to be regulated by lncRNAs. Systematic identification of testes-associated lncRNAs reveals that around 30% of lncRNAs are essential for male fertility and late spermatogenesis, with one severe case of *CR44455/6* knock-out mutant, suggesting the potential regulatory effects of lncRNAs [136]. However, *CR45362*, even though not mentioned in the systematic identification, has been reported to regulate spermatogenesis by interacting with α-Spectrin for spermatid nuclear bundling, as knock-out mutants exhibit disrupted spermatid nuclear bundling and α-Spectrin interactions [143]. An interesting case is demonstrated with *Oskar* mRNA in female oogenesis. *Oskar* is identified as a protein-coding gene, but Jenny et al. showed that it can regulate early oogenesis progress solely with the mRNA 3′UTR rescue; therefore, functioning as a lncRNA [137].

### 3.4. lncRNA in Drosophila Circadian Rhythm

The lncRNA *yellow-achaete intergenic* (*yar*) is reported to have a regulatory effect on circadian rhythm, affecting sleep behavior in *Drosophila* [139]. The conservation of *yar* is established among *Drosophila* species, and transcriptional regulation is also conserved. Mutants with a deletion in *yar* show no morphological differences, but behavioral changes in sleep regulation have been observed. Fragmented and reduced nighttime sleep is caused by *yar* mutants and results in sleep deprivation [139]. Unlike many other lncRNAs retained in the nucleus, *yar* is cytoplasmic and, therefore, may be suggestive of possible post-translational regulation for future investigation [139].

### 3.5. lncRNA and miRNA Interactions

In the previous section, we mentioned that lncRNA *msa*, lncRNA *iab-8*, and *miR-iab-8* were encoded within the same region, demonstrating some potential connections of miRNA-lncRNA interplay [133]. However, a recent study on target-directed miRNA degradation revealed that miR-310 family degradation can be triggered by the *CR43432* named *Marge* for proper cuticle development [147]. Moreover, a study on differentially expressed mRNA and lncRNA during dietary restrictions summarized a competing endogenous RNA network in *Drosophila* aging pathways [143]. The authors analyzed all the differentially expressed genes and transcripts of flies aged 7 days to 42 days, and, eventually, identified several pivotal regulatory axes and validated each through qRT-PCR [143]. The lncRNA and miRNA interactions have been reviewed for immune regulation in insect–pathogen interactions, but only miRNAs are identified in Drosophila, and no miRNA-lncRNA regulatory axis has been identified [148]. Altogether, these studies can bring fresh perspectives into the current understanding of the non-coding RNA regulatory axis within *Drosophila*.

## 4. circRNA in *Drosophila*

Another type of non-coding RNA, namely circular RNAs or circRNAs, can also have multiple diverse functions in regulating gene expression [149,150,151,152]. The circRNAs in Drosophila discussed in this section are summarised in Table 3. In general, circRNAs are stable due to their covalently closed loop structures [153]. The biogenesis of different types of circRNAs vary depending on their composition as introns or exons [153,154,155,156,157,158]. To date, circRNAs have been reported in fungi, plants, protists, and animals [159,160].

In some cases, circRNAs and related regulatory pathways are also highly conserved in animals such as between *Drosophila* and mammals [161,162,163,164]. For instance, in human and mouse cell lines, knockdown of the RNA-editing enzyme ADAR (adenosine deaminase acting on RNA) induces RNA circularisation processes. Decreases of ADAR levels in *Drosophila* due to high temperature will also lead to increased RNA circularisation [161]. Different to the linear mRNAs with the lack of 5′ and 3′ UTRs, the GW182-mediated circRNA degradation and the pathway controlling circRNA export from the nucleus are also conserved between *Drosophila* and human [165,166,167,168].

In *Drosophila*, circRNA transcripts can be identified in the embryonic stages as circular stable intronic sequences RNAs (sisRNAs), which are maternally inherited and can repress gene expression during embryogenesis [169], including *sisR-4* from *deadpan* locus triggered *deadpan* expression, forming a positive feedback loop [170].

Another circRNA known as circMbl, derived from the *muscleblind* (*mbl*) transcript, is constantly expressed throughout, except during embryogenesis and early adulthood [171]. *Mbl*, regulated by miR-277 and miR-304, is necessary for photoreceptor differentiation in *Drosophila* eyes and muscle development [38,172]. Protein MBLs may initiate the expression of their pre-mRNA [164]. With circMbl having multiple binding sites for MBL, the interaction between MBL, *mbl* pre-mRNA, and circMbl resembles a regulatory circuit when excess MBLs are translated as MBL promotes the biogenesis of circMbl, which acts as a protein sponge to MBLs [164]. Moreover, circMbl encoding-proteins in fly head extracts are enriched in synaptosomes and modulated by starvation and FOXO, whereas the MBL, circMbl, and *mbl* isoforms are expressed in a tissue-specific manner [173]. Knockdown of circMbl results in abnormal developmental phenotypes, such as high lethality and muscular defects [174].

During neuronal development in *Drosophila*, circRNA *Ect4-derived immune suppressor* (*Edis*) has been found to be essential, as *Edis* depletion shows wiring defects of the neuromuscular junctions and mushroom body neuronal development [175,176]. The circRNA encoded peptide Edis-p can also block Relish from activating the immune deficiency (IMD) pathway of the immune system, whereas Relish binds to the zinc finger transcriptional factor *castor* which promotes and up-regulates *castor* expression that functions in central nervous system development [175,176]. Furthermore, previous studies have also identified that circRNAs were abundantly localised in the *Drosophila* brain and central nervous system and would accumulate with aging [177,178,179].

A circRNA circSfl encoded by *the sulfateless* gene also produces peptides, and the overexpression of circSfl was found to extend the lifespan of around 15% of female flies [180]. Since there were less overall circRNAs accumulated in the brain of insulin *dilp* mutants through aging, and circSfl up-regulation in median-neurosecretory-cell-ablated flies was dFOXO-dependent, a relationship between insulin signalling pathways, circSfl, and aging has been proposed [180].

**Table 3:** Summarisation of circRNAs reported with their validated targets, function, and hormonal and metabolic regulations according to their references.

circRNA	Validated Targets	Function, Hormonal and Metabolic Regulations	References
*sisR-4*	*deadpan*	Embryogenesis; positive feedback loop formation	[168,169]
*circMbl*	*muscleblind*	Eye and muscle development; negative feedback loop formation	[163,173]
*Edis*	*castor*	Neuromuscular junctions; mushroom body neuronal development; IMD pathway	[175,176]
*circSfl*	N/A	Increased female lifespan; insulin pathway; aging	[180]
*circBoule*	*Hsc4, Hsp60C*	Male fertility due to heat-stress	[181]

Last but not least, in mature adults, circBoule is produced from the conserved reproductive gene *Boule* and can regulate heat-shock proteins to gradually decrease protein levels once exposed to a higher temperature. Loss of circBoule resulted in reduced male fertility in flies under a heat-stress environment [181], and such stress-induced fertility decline phenomena can also be found in humans. The above examples highlighted the importance of functional roles of circRNAs in *Drosophila*.

## 5. Conclusions and Future Perspectives

In this review, we have discussed miRNA, lncRNA, and circRNA regulation on development and metamorphosis, ecdysteroid and sesquiterpenoid systems, nutrition metabolism, sexual development, lifespan and viability, and circadian rhythm. Among all the pathways, some miRNAs have played multiple roles and served as crosstalk key components between pathways (Figure 1). Many studies start with the identification of expression profiles, lead with the miRNA target predictions, and result in mutant validations. Such comprehensive analysis can support the findings with a detailed understanding of the underlying mechanisms. Even with the global identification of miRNA-mRNA and the genome-wide lncRNA and circRNA identifications [99,141], validations of all the in silico predictions are still required to further understand the underlying mechanisms in ncRNA regulation, especially for their crosstalk in hormonal and metabolic pathways in *Drosophila*.

Another aspect is the potential of micro-peptide encoding ncRNAs. As mentioned previously, pri-miR-8 has been reported to encode for a small ORF that encodes for a miPEP-8, which can regulate the expression of hundreds more genes and will form a regulatory loop with miR-8 [182]. Similar ORFs have also been reported by other ncRNAs, such as lncRNA-MSA, *miR-14*, and many more micro-peptides encoded in lncRNAs and circRNAs [145,183,184]. How miPEPs may have regulatory effects on the hormonal and metabolic systems and whether these miPEPs may also be regulatory targets for considerations of pathway disturbances such as insecticides all require further investigation in the future.

## Figures and Tables

**Table 2:** Summarisation of lncRNAs reported with their validated targets, function, and hormonal and metabolic regulations according to their references.

lncRNA	Validated Targets	Function, Hormonal and Metabolic Regulations	References
Development, metamorphosis, and ecdysteroid hormone system			
*iab*	*Ubx*, *Abd-A*, *Abd-B*	Spatiotemporal expression pattern in development; abdomen segmentation	[127]
*acal*	N/A	JNK signalling pathway; dorsal closure	[128]
*CR33938*	N/A	Leg development	[129]
Nutrient metabolism and aging			
*IBIN*	N/A	Enhanced carbohydrate metabolism and reduced amino acid metabolism gene clusters	[130]
*IRAR*	*IR*	Nutrient-sensitive expression differences	[131]
Sexual development			
*msa*	N/A	Accessory gland development in males; cell morphology and male fertility	[132]
*iab-8*	*Hox* genes	Male and female fertility	[133]
*roX1, roX2*	MSL protein complex	Increased X-linked gene transcription; hyperactive X chromosome in males	[134,135]
*CR44455/6*	N/A	Male fertility and late spermatogenesis	[136]
*CR45362*	α-Spectrin	Spermatid nuclear bundling	[137]
*Oskar*	*mRNA-Oskar*	Female oogenesis	[138]
Circadian rhythm			
*yar*	N/A	Circadian rhythm; sleep	[139]
